# An Empirical Study on Talent Management Strategies of Knowledge-Based Organizations Using Entrepreneurial Psychology and Key Competence

**DOI:** 10.3389/fpsyg.2021.721245

**Published:** 2021-10-07

**Authors:** Mengqiong Liu

**Affiliations:** School of Economics and Management, Ningxia University, Yinchuan, China

**Keywords:** entrepreneurial psychology, key competence, knowledge organization talents, management strategy, training mechanism

## Abstract

The empirical study reported here aims to improve the effectiveness of knowledge-based talent management in science and technology enterprises and promote the stable development of enterprises. First, the impact of entrepreneurs’ psychological cognition and personal characteristics on entrepreneurial activities is analyzed based on entrepreneurial psychology. Then, the theory of key competence is introduced to study the management mode of knowledge-based talents. The advantages of talents in enterprises are sorted out through constructing the key competency model to manage talents efficiently. The technology-based enterprise M is taken as an example for analysis by the key competence model to obtain 18 key capability indexes. Through the principal component analysis of 255 employees’ survey results, finally, four factors are extracted (business execution ability, team cooperation ability, strategic thinking ability, and management decision-making ability), which can reflect 68.92% of the total key competence. The average values of “business execution ability” and “team cooperation ability” in the first-level dimension of key competence index are 4.14 and 4.24, respectively, which can be regarded as the essential key competence. The investigation results of the academic qualifications of staff of M indicate that 6% of employees have a doctorate, 38% have a master’s degree, 37% have a Bachelor’s degree, and 19% have a junior college degree or below. Moreover, knowledge-based employees are basically satisfied with the organization and management of the company, but they are dissatisfied with the training mechanism and promotion mechanism. Therefore, enterprises should pay attention to the psychological needs of knowledge workers and the innovation of talent management. The research results are of significant value for science and technology enterprises to absorb and retain knowledge-based talents and promote the common development of enterprises and employees.

## Introduction

With the continuous development and deepening of mass entrepreneurship and innovation, the policy support from the state for entrepreneurs has driven entrepreneurial atmosphere of the whole market, and more groups choose to participate in entrepreneurial activities (Wardi et al., 2018). In essence, entrepreneurs are innovators creating appropriate response measures in the face of changing economic environment, and driving economic growth in the process of innovation (Kadwa and Barnard, 2019; Semin and Kislitskiy, 2020). From the perspective of entrepreneurs, the key to the success of entrepreneurial activities is to create new products and exploit new markets. Compared with other general organizations, entrepreneurial organizations pay more attention to innovation activities (Xiong et al., 2020). However, from the actual situation, the success rate of all entrepreneurial enterprises in China is less than 50% in 5 years, and the situation is deteriorating year by year. Usually, the initial stage of newly start-ups is the period with the highest failure rate. Entrepreneurs have to not only face the chaotic and unknown market trend, but also deal with the threats of various risks. Therefore, the elementary skill that entrepreneurs need to master in the early stage of a start-up is opportunity identification (Olugbola, 2017; Schmitt et al., 2018; Asante and Affum-Osei, 2019). The inherent characteristics of entrepreneurs play a significantly important role in the identification of entrepreneurial opportunities, as well as the organizers and leaders undertaking the whole entrepreneurial activities. Moreover, entrepreneurial activities is independent behavior, and the psychological activity of entrepreneurs is also a critical branch of research on entrepreneurial behavior. In fact, key abilities are of vital importance for individuals throughout their career, in part because the key ability can distinguish common professional practical skills from knowledge and ability that are not directly related to common skills. Besides, key abilities enable a person to make judgments and choices under different circumstances or on various missions. Moreover, the key ability can also evolve into practical ability to cope with various changes in the process of career development.

Under the background of the knowledge economy, increasing entrepreneurs get down to starting science and technology enterprises (Hurel and Lobato, 2018). As innovative and pioneering enterprises with high growth and high risk, science and technology enterprises mainly rely on high-tech research and development (R&D) talents. As of February 1, 2020, there were 3.4 million science and technology enterprises in existence in China. Among them, 1.75 million enterprises were newly established in the last 3 years, primarily in Guangdong, Beijing, Jiangsu, Shanghai, and Hangzhou. For science and technology-based start-ups, knowledge is an elementary factor to stimulate continuous innovation and maintain market competitiveness. Knowledge-based talents can bring added value to products through personal thinking, judgment, creativity, and design (Kumar, 2017; Makarem et al., 2019; Alcívar Mero et al., 2020). Knowledge-based employees play an increasingly crucial role in modern enterprises, especially in science and technology enterprises. How to manage knowledge-based talents scientifically and bring value to the enterprise is a practical problem that must be considered (Löfsten et al., 2020). In this process, the personal development goals of knowledge-based talents must always be consistent with the enterprise’s strategy, to avoid brain drain and maximize the economic benefits of talents (Chen, 2019; Chen et al., 2019; Chen and Shen, 2020).

The comprehensive ability and personal quality of the talent team in the enterprise determine whether the enterprise can continue to develop. Key competence contains knowledge and abilities that are not directly related to common skills, which can be transformed into practical abilities to cope with various changes in the process of career development. Therefore, the key competence of employees will directly affect the overall development of the organization (Liu and Chen, 2021). The human resource management of science and technology enterprises has broken the conventional management mode, and the realization of human resource management of knowledge-based employees is a valuable research direction. First, from the perspective of psychology, the impact of entrepreneurs’ psychological cognition and personal characteristics on entrepreneurial activities is analyzed. With science and technology enterprises as an example, the role of key competence in talent development and talent management is discussed. On this basis, the talent management model is constructed. Then, the technology-based enterprise M is selected as the research object for the case study to investigate its current situation of talent management and propose targeted talent management strategies, to use and manage talents rationally and ultimately enhance the economic benefits of enterprises. The innovation of this work lies in adjusting the competencies to provide guarantee for the effective implementation of human resource management strategy and enterprise strategy. Through competencies adjustment, enterprises can conduct an immediate and accurate analysis of talents during rapid development, improve the talent structure in real-time, ensure the continuous supply of talents in key positions, and realize the fast expansion of enterprises through the rapid replication of talents. The innovation also lies in the talent development plan based on analyzing the talents suitable for the development of enterprises through the key competence model, facilitating the overall talent management of enterprises.

## Materials and Methods

### Cognition and Personality Characteristics of Entrepreneurs From the Perspective of Psychology

The primary mission of start-ups is to create products and values that users really need. The traditional education system and career system have instilled habitual thinking in young entrepreneurs, that they should deliberately cater to the preferences of the mainstream system to achieve a certain goal (Simpong et al., 2018). Consequently, entrepreneurs attach more importance to understanding the rules, meeting the standards of these systems, and achieving excellent peripheral management. The energy of entrepreneurs may shift from building the core product to constantly meeting the external motivation (Demetry, 2017). Therefore, grasping psychological cognition in the process of entrepreneurship is the premise of realizing the reasonable planning of entrepreneurship (Zhong et al., 2020).

The essence of cognition is the perception, memory and thinking of individuals, while cognitive psychology focuses on explaining the mental process when one interacts with people, things, and objects around. From the perspective of cognitive psychology, entrepreneurs should develop the thinking logic, entrepreneurial perception, and alertness of entrepreneurs (Castellano et al., 2017; Schermuly et al., 2021). The research shows that there are great differences in psychological cognition between entrepreneurs and non-entrepreneurs for entrepreneurship (Garbuio et al., 2018). These differences are manifested in many aspects, such as property rights protection, special skills in planning cognition, attention and self-motivation in willingness cognition, and ability-opportunity fitness and professional knowledge in ability cognition. Thus, the cognitive structure reflects the individual’s views on opportunities, risks and interests, and the cognitive process comprehensively shows the individual’s information processing ability. Based on the theory of psychological cognition, it is feasible to combine the mental and behavioral aspects of entrepreneurs to explain the related phenomena of entrepreneurial activities (Fuller et al., 2018; Pejic Bach et al., 2018).

Entrepreneurs usually have prominent adventurous spirit, achievement demand, and self-control ability. The typical explicit characteristics of entrepreneurs include optimism, leadership, perseverance, sense of responsibility and so on. The cultivation and improvement of entrepreneurs’ social and communication skills is largely driven by their psychological capital. Stable and solid psychological capital is also conducive to the entrepreneurship result. Entrepreneurs with positive psychological capital can play a significant psychological advantage when they set goals, grasp entrepreneurial opportunities, and face entrepreneurial risks. This psychological advantage will promote the development of various entrepreneurial activities and enhance the overall entrepreneurial performance of enterprises.

It is of paramount importance for enterprise management to break through the “prisoner’s dilemma” in team psychology in entrepreneurial activities. In other words, team members need to unite, trust each other, communicate frequently and stick to the contract (Giedraitis et al., 2017). Therefore, an entrepreneur must have the ability to adjust the personal style and needs of team members to form the cohesion within the group and make everyone willing to work hard for the same cause. The entrepreneurship progress is not all plain sailing. Hence, a successful entrepreneur must have a strong psychological quality, rigorous and comprehensive analytical skills, and unwavering courage and determination to complete the major decisions of the enterprise (Feng and Chen, 2020).

### Talent Management Based on Key Competency Model

**FIGURE 1:**
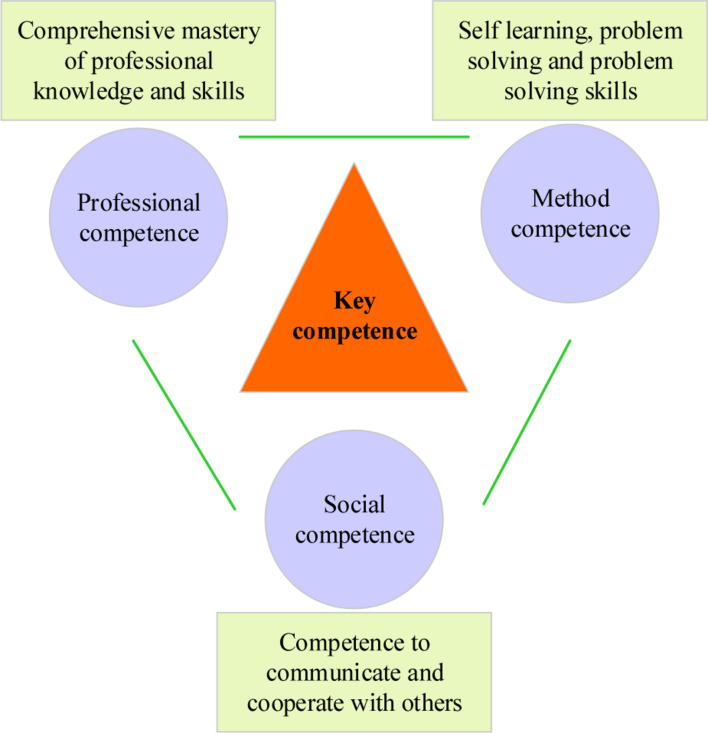
Three elements of professional action ability based on key competence.

The German social educationist D. Mertens put forward the concept of key competence in 1974. The key competence is defined as knowledge, ability and skill that are not directly related to general professional practical skills, but the ability to be competent in various unforeseen changes in life in different situations. Since then, great changes have taken place in the requirements of vocational education in Germany on the cultivation of workers’ vocational competence (Lahn and Nore, 2019; Wild and Heuling, 2020). With the change of social and technological structure, the key competence has also developed extensively in social practice. Until the 1980s, the concept of professional action ability with key competence as the core was formed, consisting of three elements, namely professional competence, method competence, and social competence. Figure 1 shows the three elements of professional action ability based on key competence.

**FIGURE 2:**
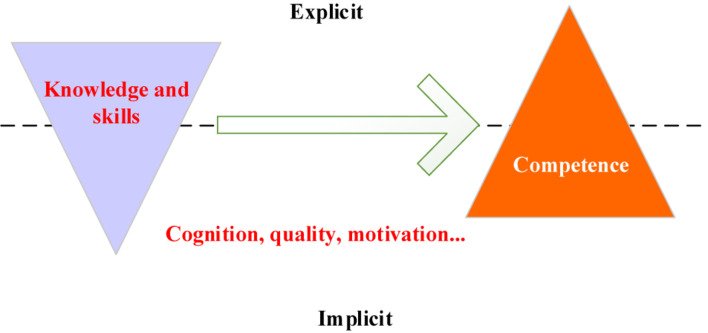
Comparison of talent selection modes.

Traditional vocational training pays more attention to the cultivation of professional skills. However, with the progress of social industrialization, to meet the needs of enterprises for knowledge-based talents, a competency-based education quality concept has gradually been established, focusing on the cultivation of key competences (Ewing, 2017; Saputra et al., 2018; Nurtanto et al., 2020). German dual system universities adopt professional competence, method competence, and social competence as support to cultivate students’ outstanding professional ability, where students should master professional knowledge, as well as method competence and social competence. In short, the connotation of key competence can be summarized as follows: (1) the competence in understanding and mastering technology; (2) the competence in making decisions; (3) the competence in solving problems independently; (4) the awareness of quality; (5) the competence in cooperation; (6) the awareness of environmental protection; (7) the sense of social responsibility. For science and technology-based enterprises, under the guidance of this competence-based view, the exploration and cultivation of key competences of knowledge-based talents is a necessary condition for the development of technological products (Hsieh et al., 2019). The current talent selection mode of Chinese enterprises presents an inverted triangle, as shown in Figure 2, lacking consideration of the psychological characteristics of talents.

According to the theoretical framework of the competence model of Shanghai Bote Management Consulting Co., Ltd., complete competency includes four parts: background conditions, professional knowledge, key competence, and internal drive. From the perspective of practical experience, the background conditions mostly involve the experience required by the job position, which is usually linked with the post experience measures. The clearest path for professional knowledge expansion is “curriculum + practice + guidance.” The ultimate expectation of key competence from enterprises is that employees can transform their key competences into routine performance, and continuously show, hone and improve them in daily work (Cai and Jun, 2018; Fillol et al., 2019). For organization managers, the specific measures to improve the key competences of employees need to be combined with the actual situation of the enterprise, especially the characteristics of the employee team. The strength of employees’ key competence directly affects the realization of employees’ competence, or the conversion of employees’ potential performance into actual performance.

The key competence model is an effective management tool to identify personal knowledge, skills and quality of employees, and the evaluation results based on the model can result in a positive impact on employees’ personal performance and enterprise development (Pascual-Fernández et al., 2021). Generally, the competences covered by key competences should be evaluable, observable, and developable, including unique competence, general competence, and transformable competence. The management of enterprises requires the coordinated development of every employee. Constructing the key competence model is conducive to the understanding of the strategic needs of the enterprise by employees, and improve their personal competence according to the needs, to promote the improvement of enterprise performance (Deng et al., 2021).

### Construction of Talent Management Model in Knowledge-Based Organization

For a knowledge-based organization, knowledge-based employees are the core elements of its production, innovation and management. The competition between enterprises is actually the competition in knowledge-based employees. From the perspective of employees, knowledge-based employees have higher education level and higher pursuit of knowledge (Dahshan et al., 2018; Sehhat and Afsharian, 2020). They are more enthusiastic about innovative work to achieve self-worth in the work, instead of the repetitive operation of ordinary work, and correspondingly some knowledge-based employees and enterprises maintain a relationship of mutual-cooperation and synchronous improvement. Moreover, with the advent of the Internet plus era, new technologies such as big data and artificial intelligence emerge endlessly. Knowledge-based employees are more inclined to take the initiative to innovation and pay more attention to the moral encouragement of enterprises in technological innovation.

When enterprises evaluate knowledge-based employees, the specific content will change according to different assessment purposes and assessment scope (Fernandes et al., 2019; Rajabpour and Chartab Moghadam, 2019). Considering the particularity of knowledge-based talents, multi-layer fuzzy comprehensive evaluation method is selected to improve the accuracy of talent management evaluation. First, the work performance of knowledge-based employees needs to be comprehensively evaluated from many aspects, including work attitude, performance, and political performance. All the included evaluation elements constitute the final system set, which is recorded as:


(1) $$U=\left\{u_{1},u_{2}\,\ldots \,u_{n}\right\}$$

The evaluation level setting varies with different evaluation indexes. The evaluation sets composed of different decisions can be expressed as:


(2) $$V=\left\{v_{1},v_{2}\,\ldots \,v_{n}\right\}$$

Generally, in the evaluation set, each element plays a different role in the overall evaluation, and the overall evaluation result largely depends on the weight distribution of each factor in the overall evaluation. Therefore, it is necessary to determine the weight distribution among each factor, which belongs to a fuzzy vector on the element set *U*.


(3) $$A=\left\{a_{1},a_{2}\,\ldots \,a_{n}\right\}$$

In Equation 3, _*ai*_ represents the weight of the *i*-th element, and ${}_{\sum_{i=1}^{n}a_{i}\;\;=1}$.

For the *i*-th index, the membership degree of each evaluation is a fuzzy subset of the evaluation set *V*.


(4) $$R_{i}=\left\{r_{i\,1},r_{i\,2}\,\ldots \,r_{i\,n}\right\}$$

Assume there is a fuzzy relation from *U* to *V*:


(5) $$R={\left(r_{i\,j}\right)}_{n\times m}$$

Then, a fuzzy transformation can be obtained by *R*.


(6) $$T_{R}:F\,\left(U\right)\to F\,\left(V\right)$$

Finally, the overall evaluation results can be obtained according to Equation 7.


(7) B=A×R

If the multi-level fuzzy evaluation involves an excess of indexes in the actual assessment, it will be difficult to determine the weight distribution in this case. Even if the weight allocation is determined, due to the normalization condition, the final weight of each element will be smaller. At this time, the two-level fuzzy evaluation method is used to divide more levels. First, the element set *U* is divided into *m* sub factor sets {*U*_1_,*U*_2_...*U*_m_} according to a specific attribute. Moreover, it satisfies that:


(8) $$n_{1}+n_{2}+\ldots \,n_{m}=n$$

(9) $$U_{1}\cup U_{2}\,\ldots \,U_{m}=U$$

Each element set _*U_i_*_ is evaluated. Denote _*R_i_*_ as the single factor evaluation matrix, and the first level evaluation vector can be recorded as:


(10) $$B_{i}=A_{i}\times R_{i}=\left(b_{i\,1},b_{i\,2}\,\ldots \,b_{i\,x}\right)$$

Each element set _*U_i_*_ is regarded as an element, as shown in Equation 11.


(11) $$R=\left[\begin{array}{c} B_{1}\\ B_{2}\\ \ldots \\ B_{m} \end{array}\right]=\left[\begin{array}{c} b_{11}\,b_{12}\,b_{1\,x}\\ b_{21}\,b_{22}\,b_{2\,x}\\ \ldots \;\ldots \;\ldots \\ b_{m\,1}\,b_{m\,2}\,b_{m\,x} \end{array}\right]$$

The proposed talent management model based on fuzzy evaluation method can not only evaluate and rank the employees according to the overall score, but also evaluate the level of employees according to the principle of maximum membership. Furthermore, the normalization is performed for a clearer employee evaluation results of talent management model.

### Empirical Analysis – Taking Technology-Based Enterprise M as an Example

The technology-based enterprise M is taken as the research object to investigate the key competence indexes of the enterprise. Then, based on the questionnaire data, a targeted enterprise key competence model is constructed through factor analysis.

The technology-based enterprise M was founded in 2000, initially to extract the effective components of agricultural products to develop natural pigments as the main business. With the continuous development of the enterprise, the company has successfully established its own brand, and its products of pigment and essential oil have been unanimously recognized in the industry. At present, the enterprise has also transformed into a company integrating research, development, production, planning, and marketing. The enterprise M has 270 members of staff, and the high-tech innovation is mainly carried out by the knowledge-based talents of the R&D center and the testing center. The R&D center has 35 industry experts as R&D project leaders, and the testing center has 65 professional and technical talents with a high knowledge level.

The expert group composed of experts, the enterprise management team, and department managers decide the key competence indexes of the enterprise M through discussion according to the development needs of the company. There are 18 elementary indexes of the key competence model. Then, a questionnaire survey is carried out on 270 employees in the enterprise M based on the 18 indexes. The 5-point Likert scale is used to rate the importance of each index, and 5 points means “very critical” while 1 point represents “not critical at all.” After sending and collecting 270 questionnaires, there are 255 valid questionnaires recovered, and the efficient rate is 94.4%. The SPSS 20.0 software is used to test the reliability of the questionnaire, and the model is constructed by factor analysis.

## Results and Discussion

### Determination of Key Competence Elements and Model Construction of M Enterprise

**FIGURE 3:**
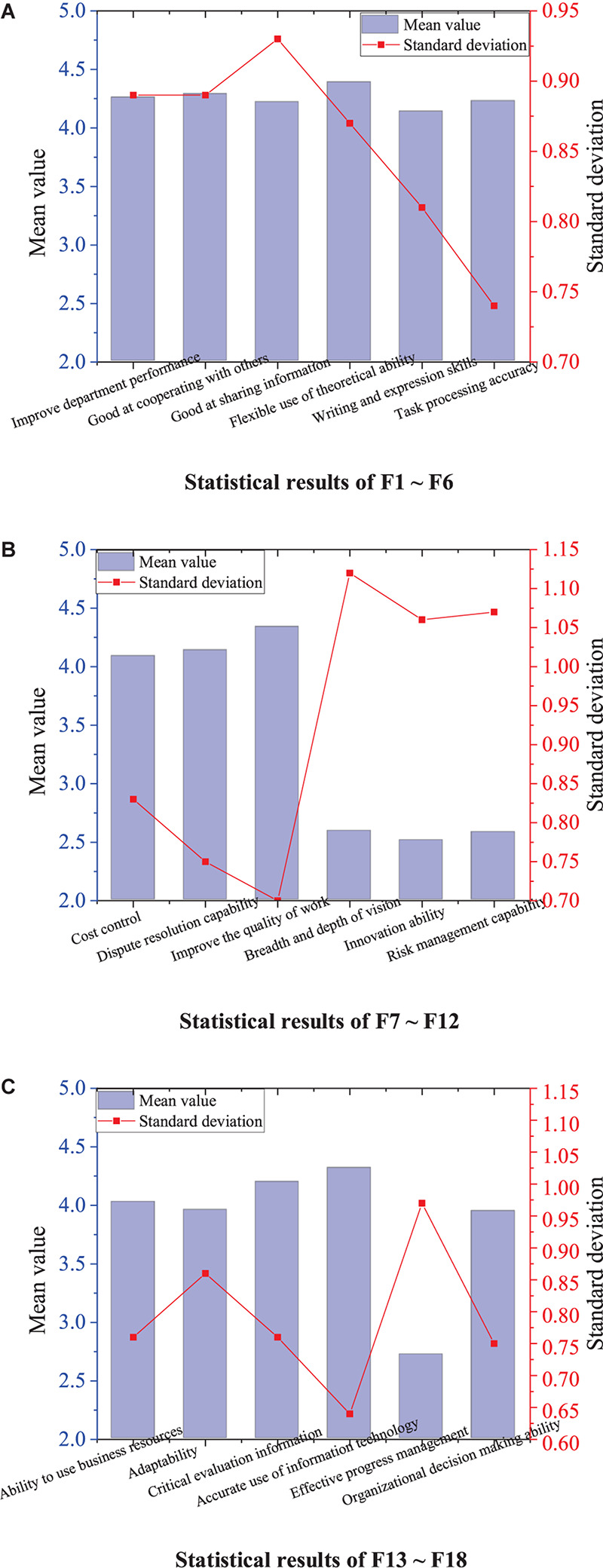
Mean and standard deviation statistics of 18 indexes. **(A)** Statistical results of F1–F6. **(B)** Statistical results of F7–F12. **(C)** Statistical results of F13–F18.

Through the discussion by the expert group, the enterprise M has established 18 key competence indexes that the enterprise needs at present. The questionnaire data is calculated to get the mean and standard deviation of each index. Figure 3 presents the specific results. Among them, Index F4 (flexible use of theoretical ability) gets the highest score of 4.39, and Index F11 (innovation ability) obtains the lowest score of 2.51. The results suggest that the scores of all the indexes are higher than the medium level, which confirms the importance of these 18 indexes. Then, the reliability of 18 indexes is analyzed, and the Cronbach’s alpha coefficient is 0.745, indicating that these 18 indexes have significant consistency.

Before factor analysis, the Kaiser-Meyer-Olkin (KMO) test and sphericity test of Bartlett are performed. The results show that KMO = 0.892, Sig. = 0.000, indicating that these indexes are suitable for factor analysis. By principal component analysis of 18 key competence indexes, four indexes are extracted, which can reflect 68.92% of the total information of key competence. According to the rotating component matrix illustrated in Table 1, the initial 18 variables are divided into four comprehensive indexes.

**TABLE 1:** Statistics of mean value and standard deviation of 18 index elements.

Factor index	Ingredient			
	1	2	3	4
F1. Improve department performance	0.333	0.605	–0.294	–0.101
F2. Good at cooperating with others	0.243	0.621	–0.261	–0.195
F3. Good at sharing information	0.138	0.871	–0.221	0.044
F4. Flexible use of theoretical ability	0.221	0.817	–0.153	0.034
F5. Writing and expression skills	0.293	0.721	–0.065	0.029
F6. Task processing accuracy	0.550	0.580	–0.048	0.021
F7. Cost control	0.595	0.365	–0.056	–0.223
F8. Dispute resolution capability	0.781	0.112	0.079	–0.191
F9. Improve the quality of work	0.761	0.235	0.000	0.072
F10. Breadth and depth of vision	–0.155	–0.287	0.725	–0.180
F11. Innovation ability	–0.104	–0.238	0.855	–0.045
F12. Risk management capability	–0.129	–0.221	0.725	–0.180
F13. Ability to use business resources	0.734	0.145	–0.303	–0.102
F14. Adaptability	0.758	0.018	–0.122	–0.005
F15. Critical evaluation information	0.722	0.256	0.173	–0.118
F16. Accurate use of information technology	0.704	0.291	–0.095	–0.076
F17. Effective progress management	–0.111	–0.079	0.183	–0.083
F18. Organizational decision making ability	–0.041	–0.041	0.017	0.904

**FIGURE 4:**
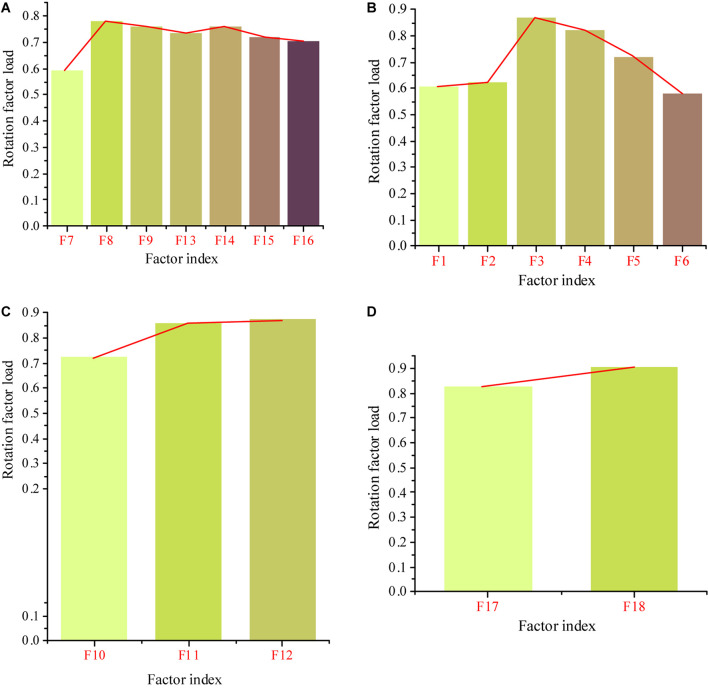
Load value of rotation factor corresponding to 4 key competence indexes. **(A)** Business execution ability. **(B)** Team cooperation ability. **(C)** Strategic thinking ability. **(D)** Management and decision-making ability.

The new four key competence indexes are renamed as business execution ability (F7, F8, F9, F13, F14, F15, and F16), team cooperation ability (F1, F2, F3, F4, F5, and F6), strategic thinking ability (F10, F11, and F12), and management and decision-making ability (F17 and F18). Figures 4A–D reveal the rotation factor loads corresponding to the four key competence indexes. The average values of business execution ability and team cooperation ability in the first level dimension of key competence indexes are 4.14 and 4.24, respectively, which can be regarded as the core of key competence. In contrast, the average value of strategic thinking ability is only 2.55, but the rotation factor load of secondary dimension index is higher, so it is also a necessary index of key competence. The average value of management and decision-making ability is in the middle level, which can be used as a relevant factor in the construction of key competence model.

### The Current Situation of Knowledge-Based Talent Management in the Enterprise M

The enterprise M is mainly responsible for the development work related to the finishing of agricultural products, and most of its employees have received professional higher education. According to the survey, many employees choose the occupation because of their professional counterparts, personal interests, or other factors. However, with the accumulation of personal working experience and the change of external environment, the market demand for knowledge-based talents is increasing, and it is not uncommon for knowledge-based employees in the enterprise M to choose job hopping. The brain drain will bring more instability factors to the development of science and technology enterprises.

**FIGURE 5:**
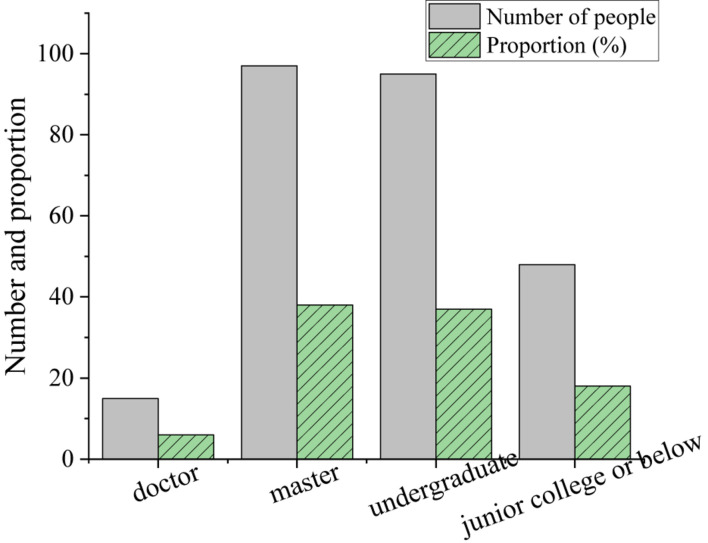
Education backgrounds of knowledge-based employees in the enterprise M.

At present, among the employees in the enterprise M, 6% are doctors, 38% are masters, 37% are Bachelors, and 19% are junior college graduates or below. Figure 5 shows the education backgrounds of knowledge-based employees in the enterprise M.

**FIGURE 6:**
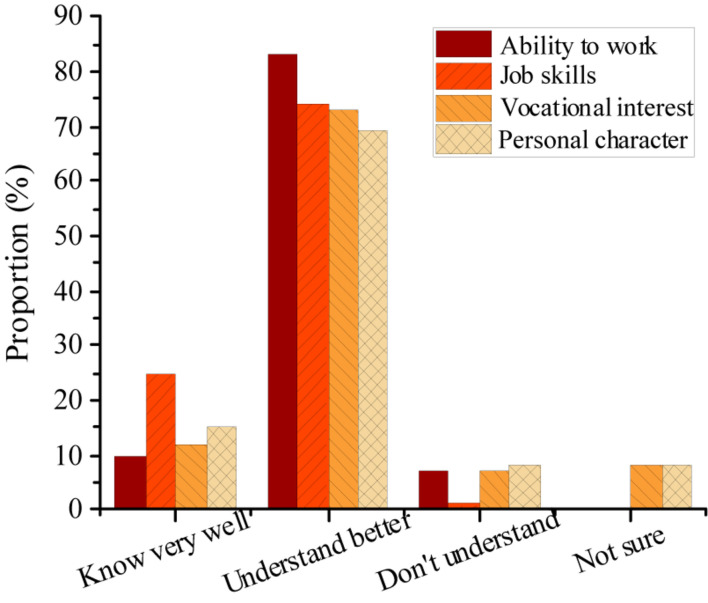
Knowledge-based employees’ understanding of their own situation in the enterprise M.

**FIGURE 7:**
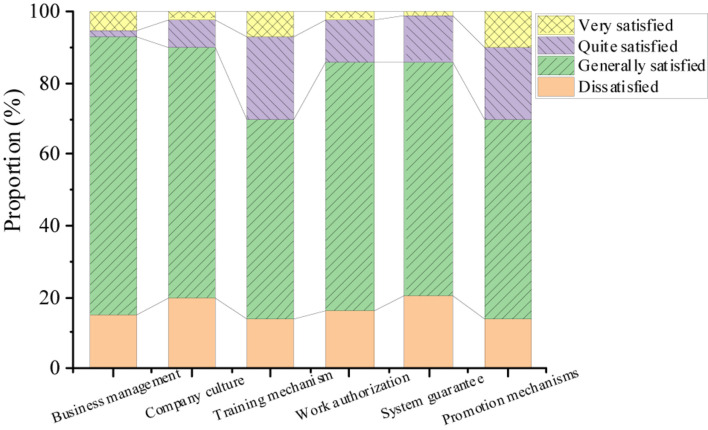
Knowledge-based employees’ satisfaction with the external environment of the enterprise M.

Figure 6 signifies the investigation results of employee’s awareness of their personal situation, including work ability, job skills, vocational interest, and personal character. More than 80% of the employees hold that they know very well about their abilities, but about 15% of the employees state that they don’t understand or they are not sure about their vocational interests and personal character. Figure 7 shows the survey results of knowledge-based employees’ satisfaction with the external environment of the enterprise M, demonstrating that employees are not satisfied with the training mechanism and promotion mechanism.

### Optimization of Management Strategies for Knowledge-Based Employees in the Enterprise M

The previous research shows that the overall evaluation of knowledge-based employees in the enterprise M on the organization and management of the company is basically appropriate, but the management strategies still need to be optimized. From the perspective of employees, knowledge-based talents usually need to acquire new knowledge and skills. However, the enterprise M is not perfect in skill training, which leads to low employee satisfaction with the company’s training mechanism. Moreover, for cost reasons, enterprises invest less time and energy in career management planning of knowledge-based employees, and the company has not formed a systematic promotion system. Based on the characteristics of knowledge-based employees, the R&D staff will consider switching the job if they bring benefits to the company but they are not rewarded with position promotion and honor.

The analysis and construction of key competence can help companies match suitable talents for key positions. The process of personnel selection, staff employment, and talent development can systematize and standardize the talent management of enterprises, which plays a guiding role in the change of the inherent concept of private enterprises and the appointment of talents. Besides, the application of key competency model plays a positive role in talent management under the background of talent shortage in the industry, conducive to the stability of talents and the healthy development of the industry. To enhance the management effects knowledge-based employees in the enterprise M, the following talent management strategies are proposed to maximize the potential of knowledge-based employees and promote the common development of enterprises and staff.

(1) Establishing knowledge-based talent management platform: it is essential to quantify the measurements of the knowledge-based talent management, including work performance, knowledge and skill level, and comprehensive ability. Work performance can measure the work performance and efficiency of knowledge-based employees, and reflect the personal value of knowledge-based talents. Knowledge and skill level demonstrates the knowledge accumulation and learning ability of employees. Comprehensive ability can show the team consciousness, social ability, and innovation ability of employees. The quantitative evaluation system can provide reference for talent management and subsequent salary and position awards.

(2) Paying more attention to knowledge-based employees: the major duty of knowledge-based employees is to create benefits for the company through scientific research and innovation. In addition to salary distribution and reward according to their work results, enterprises should also pay attention to the personal planning and inner needs of employees. Managers should improve the career planning system of knowledge-based employees, and implement it in place. Developing a complete career planning scheme for different types of knowledge-based employees can make employees clear about the future development direction and motivate employees to devote themselves to scientific research.

(3) Reforming talent management mode: the enterprise M has also carried out training activities, but failed to meet the expectations of employees. The reason is that the training generally focused on technique and business, and rarely provides the latest information on career development. In addition, because most managers use the traditional talent management methods, and ignore the combination of the enterprise’s own development strategy and employees’ personal career planning, it is easy to cause brain drain. Therefore, enterprise managers should adhere to the people-oriented principle and implement talent management based on the actual situation of the enterprise and employees.

## Conclusion

Science and technology enterprises are economic organizations which take knowledge as the main body of investment, so knowledge-based talents play an important role in the economic activities of the organization. In recent years, the management of knowledge-based talents has been a hot topic discussed by enterprises and scholars. First, based on the theory of entrepreneurial psychology, the personal characteristics and psychological cognition of entrepreneurs are analyzed. Besides, the talent management of enterprises is explored on this basis. Then, the theory of key competence is introduced, and the key competence model is analyzed to match with enterprise development strategy. Only by clarifying the key competence, can enterprises further improve the talent management system and optimize the talent structure. With the technology-based enterprise M as an example, 18 key competence indexes are determined after the discussion of the expert group. Through the questionnaire, the employees’ cognition of each index is determined. Among the first level dimension of key competence, the average values of business execution ability and team cooperation ability are 4.14 and 4.24, respectively, which can be regarded as the core of key competence. Through the survey on 255 employees’ cognition of enterprise talent management, there are still deficiencies in training mechanism and promotion mechanism of knowledge-based employees in enterprise M. Therefore, a series of optimization suggestions about talent management strategies have been proposed, focusing on the improvement of employee career management planning. This exploration is of great significance for technology-based enterprises to retain knowledge-based employees and improve the economic benefits of enterprises. The work reported here is still in the exploration stage, and there are still some deficiencies to be improved. On the one hand, there lacks comprehensive survey data, which may lead to some gaps between the conclusions and the actual needs. On the other hand, each section has not been investigated in depth for more outcomes. In future research, it is necessary to further explore more scientific indicators and model methods that are more suitable for practical needs, and pay attention to the matching of enterprise strategic planning and human resource planning.

## Data Availability Statement

The raw data supporting the conclusions of this article will be made available by the authors, without undue reservation.

## Ethics Statement

The studies involving human participants were reviewed and approved by Ningxia University Ethics Committee. The patients/participants provided their written informed consent to participate in this study. Written informed consent was obtained from the individual(s) for the publication of any potentially identifiable images or data included in this article.

## Author Contributions

The author confirms being the sole contributor of this work and has approved it for publication.

## Conflict of Interest

The author declares that the research was conducted in the absence of any commercial or financial relationships that could be construed as a potential conflict of interest.

## Publisher’s Note

All claims expressed in this article are solely those of the authors and do not necessarily represent those of their affiliated organizations, or those of the publisher, the editors and the reviewers. Any product that may be evaluated in this article, or claim that may be made by its manufacturer, is not guaranteed or endorsed by the publisher.

## References

[B1] Alcívar Mero M. J. Alarcón Chávez C. R. Ferrin Schettini H. M. (2020). Human talent and knowledge management in micro-enterprises. *Podium* 37 71–88.

[B2] Asante E. A. Affum-Osei E. (2019). Entrepreneurship as a career choice: the impact of locus of control on aspiring entrepreneurs’ opportunity recognition. *J. Bus. Res.* 98 227–235. 10.1016/j.jbusres.2019.02.006

[B3] Cai S. Jun M. (2018). A qualitative study of the internalization of ISO 9000 standards: the linkages among firms’ motivations, internalization processes, and performance. *Int. J. Prod. Econ.* 196 248–260. 10.1016/j.ijpe.2017.12.001

[B4] Castellano S. Khelladi I. Menvielle L. (2017). Unveiling the sustainable facet of the conventional entrepreneur-a cognitive approach. *Am. J. Ind. Med.* 31 434–450. 10.1504/ijesb.2017.084848

[B5] Chen M. (2019). The impact of expatriates’ cross-cultural adjustment on work stress and job involvement in the high-tech industry. *Front. Psychol.* 10:2228. 10.3389/fpsyg.2019.02228 31649581 PMC6794360

[B6] Chen M. Shen C. (2020). The correlation analysis between the service quality of intelligent library and the behavioral intention of users. *Electron. Libr.* 38 95–112. 10.1108/el-07-2019-0163

[B7] Chen J. Ye X. Chen M. Liang Y. (2019). Bibliometric analysis of the papers on urban education. *Libr. Hi Tech.* 37 894–905. 10.1108/LHT-01-2019-0009

[B8] Dahshan M. Keshk L. Dorgham L. S. (2018). Talent management and its effect on organization performance among nurses at shebin el-kom hospitals. *Int. J. Nurs. Stud.* 5 108–123.

[B9] Demetry D. (2017). Pop-up to professional: emerging entrepreneurial identity and evolving vocabularies of motive. *AMD* 3 187–207. 10.5465/amd.2015.0152

[B10] Deng X. Guo X. Wu Y. J. Chen M. (2021). Perceived environmental dynamism promotes entrepreneurial team member’s innovation: explanations based on the uncertainty reduction theory. *Int. J. Environ. Res. Public Health* 18:2033. 10.3390/ijerph18042033 33669732 PMC7921965

[B11] Ewing B. (2017). An exploration of assessment approaches in a vocational and education training courses in Australia. *ERVET* 9 1–18. 10.1080/13636820.2021.1934721

[B12] Feng B. Chen M. (2020). The impact of entrepreneurial passion on psychology and behavior of entrepreneurs. *Front. Psychol.* 11:1733. 10.3389/fpsyg.2020.01733 32793066 PMC7385187

[B13] Fernandes R. Carey D. Bortoluzzi B. McArthur J. J. (2019). Development and field testing of a multi-dimensional tool for benchmarking knowledge worker productivity. *Intell. Build. Int.* 11 227–247. 10.1080/17508975.2019.1674625

[B14] Fillol A. Lohmann J. Turcotte-Tremblay A. M. Somé P. A. Ridde V. (2019). The importance of leadership and organizational capacity in shaping health workers’ motivational reactions to performance-based financing: a multiple case study in Burkina Faso. *Int. J. Health Policy Manag.* 8 272–279. 10.15171/ijhpm.2018.133 31204443 PMC6571493

[B15] Fuller B. Liu Y. Bajaba S. Marler L. E. Pratte J. (2018). Examining how the personality, self-efficacy, and anticipatory cognitions of potential entrepreneurs shape their entrepreneurial intentions. *Pers. Individ. Dif.* 125 120–125. 10.1016/j.paid.2018.01.005

[B16] Garbuio M. Dong A. Lin N. Tschang T. Lovallo D. (2018). Demystifying the genius of entrepreneurship: how design cognition can help create the next generation of entrepreneurs. *AMLE* 17 41–61. 10.5465/amle.2016.0040

[B17] Giedraitis A. Stašys R. Skirpstaitė R. (2017). Management team development opportunities: a case of Lithuanian furniture company. *Entrep. Sustain. Issues* 5 212–222. 10.9770/jesi.2017.5.2(4)

[B18] Hsieh P. J. Chen C. C. Liu W. (2019). Integrating talent cultivation tools to enact a knowledge-oriented culture and achieve organizational talent cultivation strategies. *Knowl. Manage. Res. Pract.* 17 108–124. 10.1080/14778238.2019.1571872

[B19] Hurel L. M. Lobato L. C. (2018). Unpacking cyber norms: private companies as norm entrepreneurs. *J. Cyber. Policy* 3 61–76. 10.1080/23738871.2018.1467942

[B20] Kadwa I. Barnard B. (2019). The impact of leadership on entrepreneurship and innovation: perceptions of entrepreneurs. *IUP J. Entrep. Dev.* 16 7–43.

[B21] Kumar A. (2017). Enhancing business performance through talent management systems and positive thought action, skills and knowledge: an empirical study in the Indian IT sector. *IJTM* 7 85–100.

[B22] Lahn L. C. Nore H. (2019). Large scale studies of holistic professional competence in vocational education and training (VET). *Case Norway. IJRVET.* 6 132–152. 10.13152/ijrvet.6.2.2

[B23] Liu Y. Chen M. (2021). Applying Text Similarity Algorithm to Analyze the Triangular Citation Behavior of Scientists. *Appl. Soft Comput.* 107:107362. 10.1016/j.asoc.2021.107362

[B24] Löfsten H. Klofsten M. Cadorin E. (2020). Science parks and talent attraction management: university students as a strategic resource for innovation and entrepreneurship. *Eur. Plan. Stud.* 28 2465–2488. 10.1080/09654313.2020.1722986

[B25] Makarem Y. Metcalfe B. D. Afiouni F. (2019). A feminist poststructuralist critique of talent management: toward a more gender sensitive body of knowledge. *BRQ Bus. Res. Q.* 22 181–193. 10.1016/j.brq.2019.04.004

[B26] Nurtanto M. Pardjono P. Ramdani S. D. (2020). The effect of STEM-EDP in professional learning on automotive engineering competence in vocational high school. *JEGYS* 8 633–649. 10.17478/jegys.645047

[B27] Olugbola S. A. (2017). Exploring entrepreneurial readiness of youth and startup success components: entrepreneurship training as a moderator. *J. Innov. Knowl.* 2 155–171. 10.1016/j.jik.2016.12.004

[B28] Pascual-Fernández P. Santos-Vijande M. L. López-Sánchez J. Á Molina A. (2021). Key drivers of innovation capability in hotels: implications on performance. *Int. J. Hosp. Manag.* 94:102825. 10.1016/j.ijhm.2020.102825

[B29] Pejic Bach M. Aleksic A. Merkac-Skok M. (2018). Examining determinants of entrepreneurial intentions in Slovenia: applying the theory of planned behaviour and an innovative cognitive style. *Econ. Res.* 31 1453–1471. 10.1080/1331677x.2018.1478321

[B30] Rajabpour E. Chartab Moghadam J. (2019). Assessment talent management strategy on job satisfaction and organizational commitment knowledge worker. *J. Evol. Equ.* 1397 109–117.

[B31] Saputra M. D. Joyoatmojo S. Wardani D. K. (2018). The assessment of critical-thinking-skill tests for accounting students of vocational high schools. *Int. J. Educ. Res.* 3 85–96. 10.24331/ijere.453860

[B32] Schermuly C. C. Wach D. Kirschbaum C. Wegge J. (2021). Coaching of insolvent entrepreneurs and the change in coping resources, health, and cognitive performance. *J. Appl. Psychol.* 70 556–574. 10.1111/apps.12244

[B33] Schmitt A. Rosing K. Zhang S. X. Leatherbee M. (2018). A dynamic model of entrepreneurial uncertainty and business opportunity identification: exploration as a mediator and entrepreneurial self-efficacy as a moderator. *Entrep. Theory Pract.* 42 835–859. 10.1177/1042258717721482

[B34] Sehhat S. Afsharian M. (2020). A talent management model for active knowledge-based companies in the agricultural sector of Guilan Province (Case of Rasht City). *IJAMAD* 10 189–206.

[B35] Semin A. Kislitskiy M. (2020). The econometric model for assessing the economic category of a Russian farmer entrepreneur in terms of the “Innovator vs. Conservative” system. *JEECAR* 7 255–266. 10.15549/jeecar.v7i3.597

[B36] Simpong D. B. Zahari M. S. M. Ahmad R. Hanafiah M. H. (2018). Indigenous entrepreneurs and the mainstream tourism industry related businesses: a case of Orang Asli in Malaysia. *MJFAS* 10 436–462.

[B37] Wardi Y. Susanto P. Abror A. Abdullah N. L. (2018). Impact of entrepreneurial proclivity on firm performance: the role of market and technology turbulence. *Pertanika J. Soc. Sci. Hum.* 26 241–250.

[B38] Wild S. Heuling L. S. (2020). How do the digital competences of students in vocational schools differ from those of students in cooperative higher education institutions in Germany. *ERVET* 12 1–18. 10.1016/j.iheduc.2016.02.002

[B39] Xiong Z. Wang P. Zhao Y. (2020). Re-innovation from failure, institutional environmental differences, and firm performance: evidence from China. *Amfiteatru Econ.* 22 197–219. 10.24818/ea/2020/53/197

[B40] Zhong H. Yan R. Li S. Chen M. (2020). The psychological expectation of new project income under the influence of the entrepreneur’s sentiment from the perspective of information asymmetry. *Front. Psychol.* 11:1416. 10.3389/fpsyg.2020.01416 32774311 PMC7381333

